# Synergistic Behavior of Polyethyleneimine and Epoxy Monomers Loaded in Mesoporous Silica as a Corrosion-Resistant Self-Healing Epoxy Coating

**DOI:** 10.1021/acsomega.2c01508

**Published:** 2022-08-30

**Authors:** Muddasir Nawaz, A. Bahgat Radwan, Pramod K. Kalambate, Wanida Laiwattanapaisal, Fareeha Ubaid, Himyan M. Akbar, R. A. Shakoor, Ramazan Kahraman

**Affiliations:** † Center of Advanced Materials (CAM), Qatar University, Doha 2713, Qatar; ‡ Department of Clinical Chemistry, Faculty of Allied Health Sciences, Chulalongkorn University, Bangkok 10330, Thailand; § Department of Chemical Engineering, College of Engineering, Qatar University, Doha 2713, Qatar

## Abstract

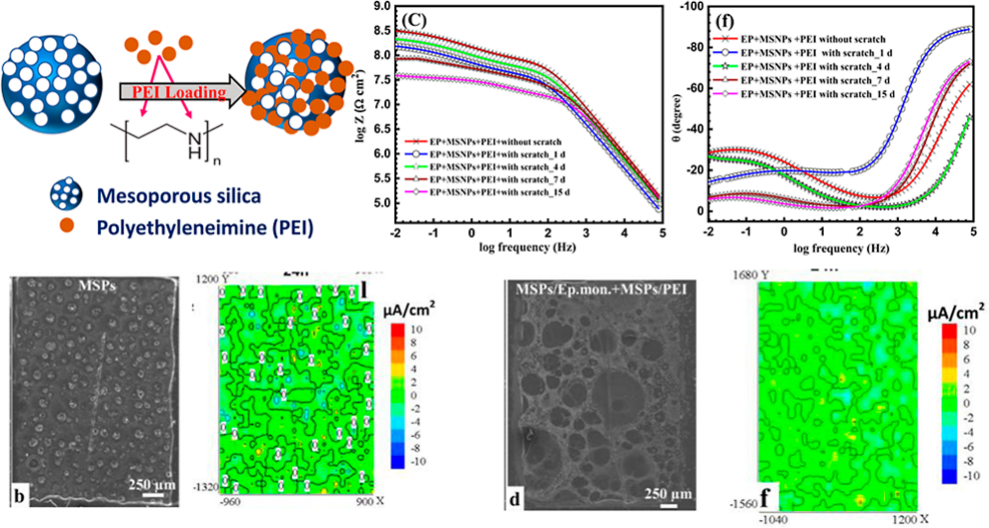

Corrosion is a significant problem and is, to a large extent, responsible for the degradation of metallic parts. In this direction, mesoporous silica particles (MSPs) were synthesized by a sol–gel technique and had an average pore diameter of ∼6.82 nm. The MSPs were loaded with polyethyleneimine (PEI) and epoxy monomers and, after that, carefully mixed into the epoxy matrix to formulate new modified polymeric coatings. The microstructural, compositional, structural, and thermal properties were investigated using various characterizing tools [Transmission electron microscopy, Fourier transform infrared spectroscopy, hermogravimetric analysis (TGA), and X-ray photoelectron spectroscopy]. TGA confirms the loading of mesoporous silica with a corrosion inhibitor, and its estimated loading amount is ∼8%. The electrochemical impedance spectroscopy properties of the reference and modified coated samples confirm the promising anti-corrosive performance of the synthesized polymeric smart coatings. Localized electrochemical tests (scanning vibrating electrode technique and scanning ion-selective electrode technique) evidence the corrosion inhibition ability of the coating, and its self-healing was also observed during 24 h of immersion. The decent anti-corrosion performance of the modified coatings can be credited to the efficient synergistic effect of the PEI and epoxy monomer.

## 1 Introduction

Corrosion prevention is an essential concern in numerous industries where metallic parts are used for different operations. Prevention of in-service parts from corrosion is an ongoing challenge, which needs immediate and regular attention. Almost all the metals and alloys instigated in oil and gas industries are susceptible to corrosion damage.^1,2^ Polymeric coatings are usually applied on metal surfaces to provide a physical barrier (passive corrosion protection) against corrosion attacks from the surrounding environment. However, the coating starts to degrade if it gets damaged, which lessens the anti-corrosion property of the coatings.^3^ Active corrosion protection can be achieved by incorporating coating with anti-corrosive agents and pigments that can leech these substances to mitigate the corrosion process. Nano-/micro-sized containers loaded with active species have turned out to be quite promising.^4−6^ These inorganic micro/nanofillers are also referred to as smart containers; by suitable designing of smart containers, the self-release of the stored active species occurs (corrosion inhibitors and self-healing agents) triggered by an external stimulus, for example, mechanical damage, pH change, light sensitivity, and so forth.^4,7^ Active corrosion protection can prolong the service life of equipment by providing an on-demand release of the inhibitor in the affected area.^8,9^ Several types of microcontainers and nanocontainers and encapsulation processes have already been used and reported in the literature, including micro- and nano-capsules,^10−12^ nanotubes,^13,14^ polyelectrolyte shell capsules,^15^ porous shell capsules,^16^ ion exchange substances,^17,18^ layered double hydroxides,^19^ and nanofiber materials.^20,21^ Porous materials used as nanoreservoirs, that can provide sustained release over a more extended period as the active species can be stored inside the porous structure.

MSPs are very attractive to use as containers for loading with corrosion inhibitors because of their high thermal stability, more surface area, higher loading capability, controllable pore diameter, and chemical inertness to organic and inorganic corrosion inhibitors.^22−24^ Despite these promising applications, only a few studies have been reported on the use of mesoporous silica as a reservoir for corrosion inhibitors. It is worth mentioning that some polymeric compounds possess superior corrosion resistance due to their strong adsorption on the metal substrate. Jianguo et al.^25^ reported that polyethyleneimine (PEI, 50 × 10^–3^ g mol^–1^) is very active as a corrosion inhibitor for steel in a H_3_PO_4_ environment. For instance, compared to polyvinylpyrrolidone, PEI is more effective as an anodic corrosion inhibitor for steel protection. Borisova et al.^26^ loaded mesoporous silica with benzotriazole (BTA) to study the protection of A2024 steel against corrosion. Feng and Cheng^8^ studied the silica (SiO_2_) nanoparticle-based polyelectrolyte nanocontainers, where BTA was loaded as corrosion inhibitors, and the nanocontainers were doped in an industry-based epoxy. Falcón et al.^3^ investigated the loading of dodecylamine in highly ordered MSPs to evaluate its anti-corrosive performance at various pH values.

The present study focuses on the design, synthesis, and performance of MSPs as a reservoir loaded with an organic corrosion inhibitor, PEI, and epoxy (EP) monomers. The MSPs incubated with EP monomers (MSPs-Ep monomers) and MSPs loaded with PEI were thoroughly mixed with EP matrix to develop smart polymeric coatings. The addition of EP monomers impregnated with MSPs helps recover the damage in the EP coating in the presence of PEI. The loading of the corrosion inhibitor and EP monomer on/inside the mesoporous silica prevents the direct interaction of the inhibitor with the EP coating due to the curing kinetics being slower than that of EPIKURE 3223 diethylenetriamine because of the lower mobility of the PEI.^27,28^ Structural, morphological, thermal, and anti-corrosion tests were conducted to assess the corrosion resistance behavior of the developed polymeric modified coatings.

## 2 Experimental Methods

### 2.1 Materials and Chemicals

Pluronic (P_123_, EO_20_PO_70_EO_20_), ethanol (laboratory reagent, 96%), and hydrochloric acid (ACS reagent 37%) were supplied by Sigma-Aldrich. Tetraethyl orthosilicate (TEOS, reagent grade 98%) was also supplied by Sigma-Aldrich (reagent grade 98%), which was used as a template for silica. EP resin (Epon 815C, bisphenol A epichlorohydrin polymer) and its curing agent (EPIKURE-3223) were obtained from the Miller–Stephenson Chemical Co., USA. Low-carbon steel plates (30 × 30 × 1.0 mm^3^) having a composition of Fe = 99.18%, C = 0.21%, Cu = 0.20%, Mn = 0.30%, P = 0.04%, and S = 0.04% utilized as substrates were ground and polished with various grit sizes of SiC papers from 220 to 1200. Afterward, steel samples were rinsed and carefully washed with distilled water and ethanol before applying coatings.

### 2.2 Synthesis of MSPs

MSPs were synthesized using amphiphilic triblock copolymer poly(ethylene glycol)-block-poly(propylene glycol)-block-poly(ethylene glycol) (EO_20_PO_70_EO_20_).^29^ In the first step, 4 g of the amphiphilic triblock copolymer was dissolved in 30 mL of deionized water and 8.7 g of 2 M HCl. The resulting mixture was kept under stirring for 5 h to attain a homogenous solution. Later, 9.4 g of TEOS was added to the homogenous solution and kept stirring. Then, the resulting gel was kept under stirring for 24 h at 35–40 °C, followed by heating at 100 °C for 18 h. The solid product obtained was then filtered and washed repeatedly with distilled water and ethanol to remove the presence of any copolymer, followed by drying at 25 °C. Calcination of the dried product was carried out at 550 °C for 6 h at a heating rate of 10 °C/min in an ambient atmosphere to remove the presence of any surfactant. The calcined product was then cooled to room temperature to get SBA highly ordered MSPs.

### 2.3 Loading of EP Monomers and PEI in MSPs

The synthesized MSPs were loaded with PEI by adding 0.5 g of MSPs in an aqueous solution (10 mg/mL) of PEI. The pH of the solution was modified to 4.0 to facilitate the efficient loading of inhibitors inside the MSPs. The resulting suspension was sonicated for 15 min and then moved to a vacuum chamber. The chamber was evacuated utilizing a vacuum pump, which helps to significantly decrease the presence of any air molecules in the internal channels of the MSPs. Then, the chamber was sealed for 6 h, allowing PEI to attain equilibrium between the inner channels of the pores and the surrounding solution. The vacuum process was followed by centrifugation, washing with distilled water, and drying overnight at room temperature. Encapsulation of EP monomers inside the MSPs was carried out by stirring a mixture of epoxy 815C and MSPs at 200 rpm for 1 h. After that, the homogeneous solution was kept for 6 h in a vacuum chamber at a pressure of 10^–5^ bar.

### 2.4 Development of Polymeric Coatings

**Figure 1:**
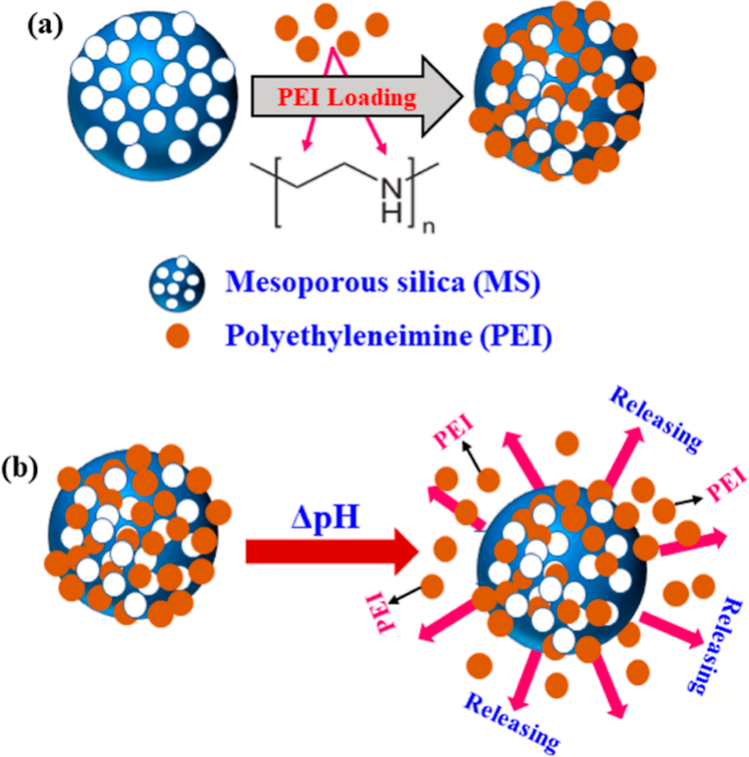
(a) Schematic representation of loading PEI in mesoporous silica and (b) schematic of the release mechanism of the inhibitor from MSPs.

Three different types of coatings were formulated for a clear comparison. EP coatings contain only MSPs. Modified coatings contain MSPs loaded with PEI and MSPs incubated with the EP monomer. As a first step, 5.0 wt % MSPs were carefully dispersed into the EP resin (815C). After stirring for 15.0 min, a hardener (EPIKURE 3223) with a 4:1 ratio was added and kept stirring for 5 min. Then, the formulated EP was coated on the surface of steel substrates by the doctor’s blade technique. The coated steel coups were kept for curing at room temperature for 1 week. Resultantly, a dry film having a thickness of ∼111 ± 5 μm was attained. A schematic diagram for PEI loaded into MSPs and the release of PEI from MSPs is shown in Figure 1.

### 2.5 Characterization

The topology of the as-prepared coatings was explored using a field emission scanning electron microscope (Nova Nano FESEM system) coupled with an energy-dispersive X-ray spectroscopy (EDS) analyzer. The microstructural features were revealed at different stages of the development, that is, as-synthesized MSPs, MSPs loaded with PEI, and MSPs loaded with the EP monomer. The surface morphology of the MSPs and loaded product was studied by transmission electron microscopy (TEM, FEI, TALOS F200X, USA). Moreover, the morphology of the developed coatings and the distribution of MSPs into the EP matrix were also investigated. Zeta (ζ ) potential of the as-synthesized MSPs and PEI-loaded MSPs was measured in deionized water at pH ∼ 7 using ζ potential equipment (Malvern, Zeta sizer, Nano ZSP, USA). Each calculated value was an average of three runs of the instrument. Fourier transform infrared (FTIR) (PerkinElmer, USA) spectroscopy was conducted in the range from 500 to 4000 cm^–1^ at a spectral resolution of 4.0 cm^–1^ to analyze the presence of functional groups. A thermogravimetric analysis (TGA) synchronization analyzer (Pyris 4000) was utilized to check the thermal stability and the quantity of the PEI corrosion inhibitor loaded inside MSPs. TGA was conducted from 30 to 600 °C at a 10 °C/min heating rate in a nitrogen atmosphere. An X-ray photoelectron spectrometer, Axis ultra DLD, was utilized to analyze the chemical composition of the developed polymeric smart coatings containing MSPs utilizing a monochromatic Al Kα source. The powder sample was spread over a double-sided carbon tape, which was placed on a sample holder and gently pressed into the tape. The sample holder was tilted to remove loose particles. X-ray photoelectron spectrometry (XPS) survey spectra were recorded with the binding energy (BE) ranging from 0 to 1200 eV. The high-resolution spectra were measured for each element at an energy step size of 0.1 eV at a pass energy of 10 eV.

The coating thickness of the coating was measured using a gauge meter (PosiTector 6000) DeFelsko (made in USA). The anti-corrosion properties of the coated steel specimens before and after the scratch were inspected at room temperature in 3.5 wt % NaCl solution by electrochemical impedance spectroscopy (EIS) analysis. A three-electrode electrochemical cell composed of the coated steel specimens as the working electrode, silver/silver chloride (Ag/AgCl) as the reference electrode, and a graphite rod that acts as a counter working electrode, respectively, was employed for EIS analysis. The electrochemical analysis was carried out in an exposed area of 2.84 cm^2^ utilizing a Gamry 3000 (30K BOOSTER potentiostat/galvanostat/ZRA, USA), in the frequency range from 0.01 to 1 × 10^2^ kHz. The EIS was recorded at OCP, and the RMS signal was 10 mV.

The evolution of localized corrosion activity for coated samples was conducted by scanning vibrating electrode technique/scanning ion-selective electrode technique (SVET/SIET) experiments carried out with applicable electronics, where current density and pH acquisition were controlled using ASET software (Sciencewares). A scratch defect (width ∼ 40 μm and length ∼ 2 mm) was monitored employing the SVET and SIET. The acquisition of SVET and SIET scanning points was performed quasi-simultaneously. SVET measurements were executed using commercial Pt–Ir microprobes (Science Products) with Pt black being deposited at the tip (final tip diameter ∼ 15 μm). A SVET microprobe was positioned 100 ± 2 μm above the surface. The vibration frequencies of the probe were 124 Hz (*Z*, vertical component) and 323 Hz (*X*, horizontal component). Only the vertical component of vibration was considered for further analysis of SVET data.

## 3 Results and Discussion

### 3.1 Structural Characterization

**Figure 2:**
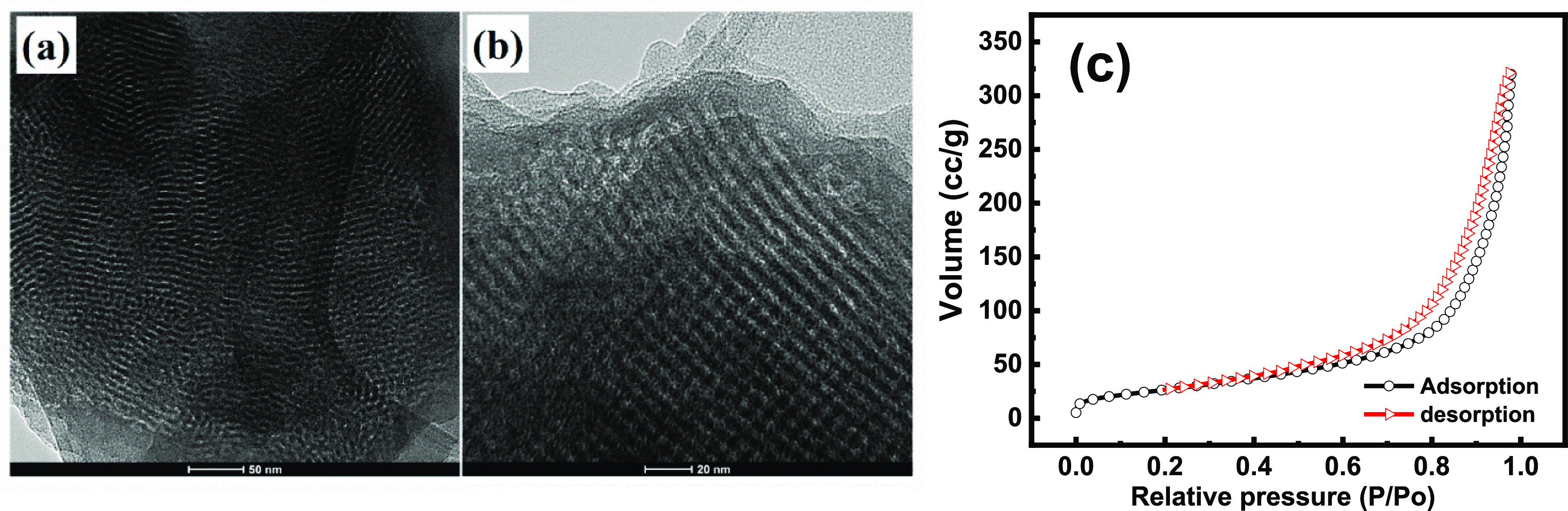
TEM images of the (a) synthesized MSPs (b) MSPs loaded with PEI and (c) N_2_ adsorption–desorption isotherms of MSPs.

The morphology of the as-synthesized MSPs loaded with PEI is depicted in Figure 2. It can be noticed from Figure 2a,b that the adopted synthesis process has resulted in the construction of MSPs with a small pore diameter. The pore diameter and the surface area of the as-prepared MSPs were explored employing the N_2_ adsorption–desorption isotherms, see Figure 2c. The determined BET surface area was found to be 102 m^2^ g^–1^ and the total pore volume was 0.5 mL g^–1^. The average pore diameter was estimated to be 6.81 nm.

### 3.2 FTIR Analysis

**Figure 3:**
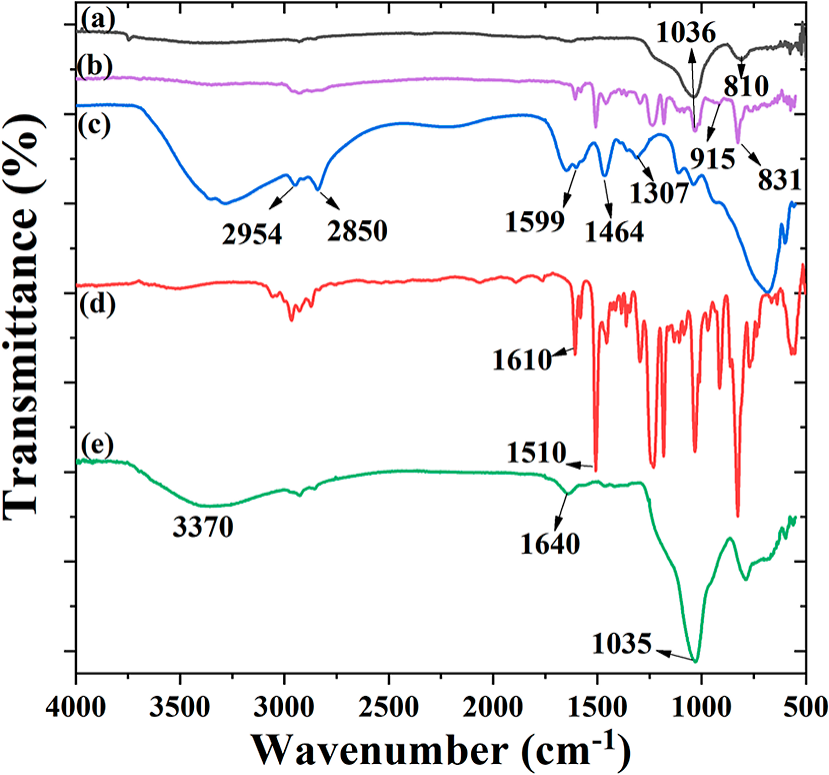
FTIR spectra of (a) MSPs, (b) EP monomers, (c) PEI, (d) MSPs-EP monomers, and (e) MSPs-PEI.

Figure 3 displays the FTIR spectrum of the Ep monomer, as-prepared MSPs, MSPs-Ep monomers, PEI, and loaded MSPs-PEI. It can be clearly observed (see Figure 3a) in spectra that the characteristic peaks of SiO_2_ present at 810 and 1040 cm^–1^ are linked with the bending and stretching vibrations of Si–O–Si bonds.^22,30^ The stretching band of C–H around 3100–2900 cm^–1^ in Figure 3b is due to the oxirane ring from EP monomers. The band between 1500–1650 cm^–1^ is due to −CH_2_ and −CH_3_ of Ep monomers. The epoxide ring was examined at 915 cm^–1^, while aromatic deformation was observed at 1036 cm^–1^. The FTIR spectrum of PEI displays a broad band at 3300–3450 cm^–1^ in Figure 3c that corresponds to N–H stretching vibrations. However, peaks located at 2850 and 2954 cm^–1^ are attributed to aliphatic CH_2_ symmetrical and asymmetrical vibration stretching of PEI.^31−33^ The observed band at 1640 cm^–1^ is attributed to the bending vibration peaks of −N–H groups in PEI.^34^ The two peaks positioned at 1599 and 1464 cm^–1^ are related to the N–H vibrations of primary and secondary amino groups, respectively. It is noteworthy that the peak at 1464 cm^–1^ is also associated with the C–H bonds. The peaks at 1307, 1042, and 1115 cm^–1^ correlate with the C–N stretching vibrations of PEI. However, in the case of MSPs loaded with PEI, the presence of a wide band is attributed to the physically adsorbed water.^35^ The characteristic peaks of Ep monomers in the region of 1600–700 cm^–1^ can also be found after loading of MSPs with EP monomers (see Figure 3d). The adsorption band of silicate from MSPs was also observed at 1640 and 810 cm^–1^ in the case of MSPs-PEI (Figure 3e).

### 3.3 TGA

**Figure 4:**
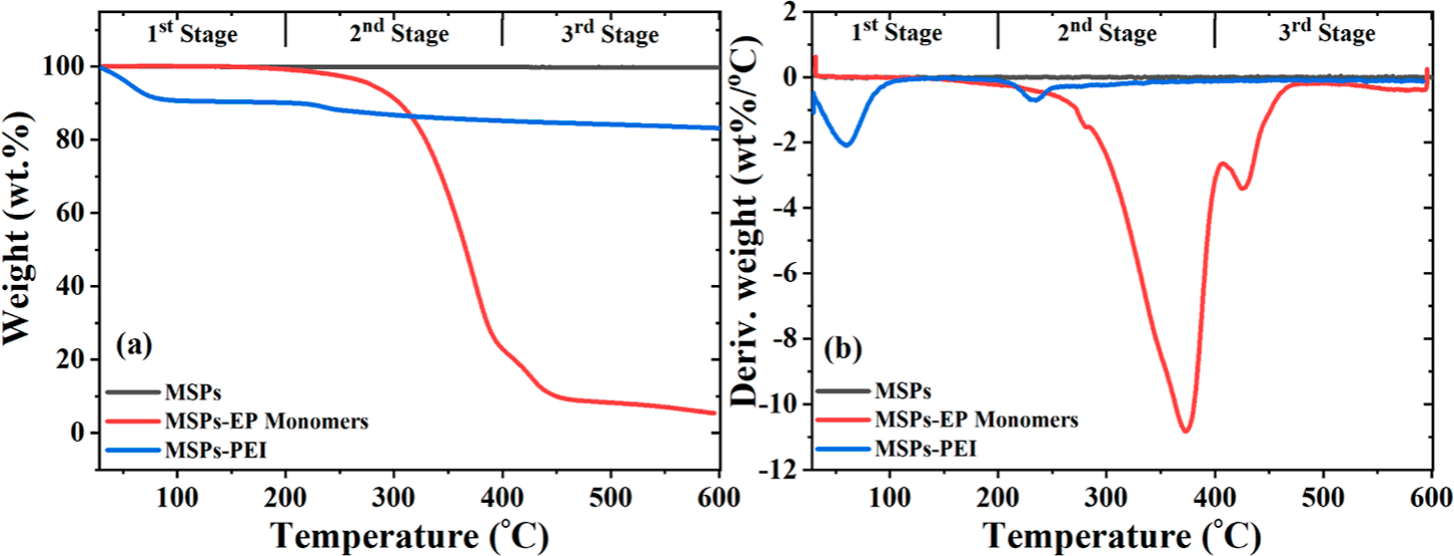
(a) TGA and (b) DTG of MSPs, MSPs impregnated with Ep monomers, and MSPs loaded with PEI.

TGA and differential thermogravimetry (DTG) profiles of the as-synthesized MSPs, MSPs-Ep monomers, MSPs loaded with PEI are represented in Figure 4. TGA of the MSPs was performed not only to estimate the loaded amount of inhibitor but to have a deep insight into the thermal behavior/stability of the modified MSPs over a range of temperature. It can be observed that the as-synthesized MSPs show superior thermal stability, and no of weight loss observed with increasing temperature til 600 °C, as revealed in Figure 4a. However, in the case of MSPs-Ep monomers, initially no weight loss was noted during the first stage, but during the second stage, sudden weight loss was observed. This is due to the breakdown and degradation of long chains of EP monomers. The larger weight loss can be ascribed to the presence of the adsorbed EP monomer on the wall of MSPs. However, in the case of PEI-loaded MSPs, two prominent weight loss stages are noticed by differential thermal analysis (Figure 4b). One weight loss dip was observed during the first stage at 58.9 °C while another was observed during the second at 234 °C. Initial weight loss during the first stage for MSPs-PEI was ∼9 wt %, which was probably due to some residual moisture content from the PEI solution, and the presence of moisture content can also be confirmed from the presence of the O–H bond in the FTIR spectra of MSPs-PEI. The weight loss is about 8 wt % (during the second stage), which is due to the thermal decomposition of the loaded PEI in MSPs.^36^ The TGA results indicate that the amount of PEI loaded into the MSPs was ∼8%.

### 3.4 ζ Potential

The ζ potential values of the as-synthesized MSPs and PEI-loaded MSPs were evaluated at pH 7. As-synthesized MSPs display a negative ζ potential of −20.8 mV. This is a characteristic feature of the MSPs as they display a negative surface charge above the isoelectric point (pH ∼ 2–3).^4^ After loading PEI into MSPs, the value of the ζ potential was shifted toward the positive side, indicating a value of 48.1 mV. This positive shift of the ζ potential is attributed to the interaction between the hydroxyl groups of MSPs and the cationic PEI. The interactions of an amine group (−NH_2_) of the corrosion inhibitor with the hydroxyl groups (OH−) of the mesoporous silica lead to the formation of Si–OH···N hydrogen bonds as shown by the XPS results. These results are consistent with the previously reported literature.^37−39^

### 3.5 XPS Analysis

**Figure 5:**
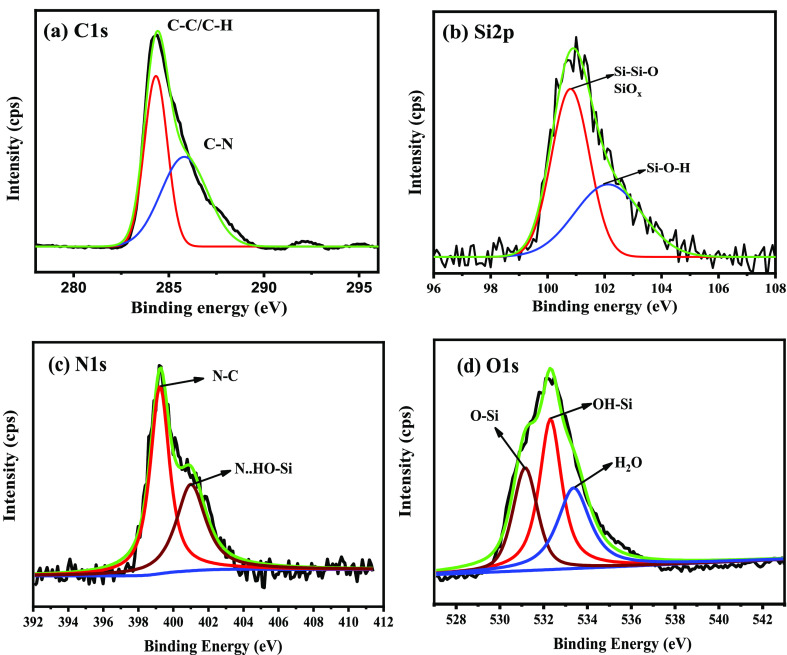
High-resolution XPS spectra of (a) C 1s, (b) Si 2p, (c) N 1s, and (d) O 1s of the PEI-loaded MSPs.

The XPS spectrum of the PEI-loaded MSPs is presented in Figure 5. The C 1s spectra are deconvoluted into two peaks of spectra, see Figure 5a. The peaks positioned at 284.4 and 285.6 eV are allied to the presence of C–C/C–H bonds and C–N bonds of PEI, respectively.^40^ The sub-peak placed at 100.8 is attributed to Si–Si–O and/or SiO_*x*_, while the sub-peak centered at 102.1 eV is accredited to Si–O–H; see Figure 5b.^41,42^ On the other hand, the sub-peak located at 399.2 ± 0.1 eV is assigned to the adsorbed PEI corrosion inhibitor on the outer surface of mesoporous silica due to C−N bonds. Nevertheless, the peak placed at 400.2 ± 0.1 eV can be attributed to the interactions of an amine group (−NH_2_) of the corrosion inhibitor with the hydroxyl groups (OH^–^) of the mesoporous silica, leading to the formation of Si–OH···N hydrogen bonds; see Figure 5c.^41^ Moreover, the binding energies of O 1s identified at 531.5, 532.3, and 533.7 eV correspond to Si–O/Si–OH and adsorbed water, respectively; see Figure 5d.^43^

### 3.6 Characterization of the Modified Coatings

#### 3.6.1 SEM Analysis

**Figure 6:**
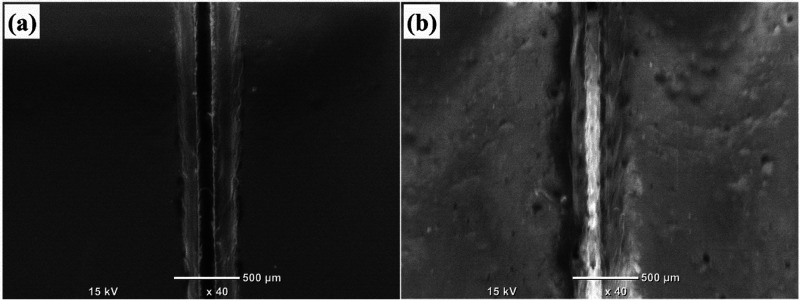
SEM images of the EP coating containing MSPs loaded with PEI and MSPs loaded with EP monomers after (a) scratch on day 1 and (b) self-healing on day 7.

Figure 6a,b exhibits the SEM images of the scratched (width ∼ 101 μm) modified coating containing PEI-loaded MSPs that was exposed to 3.5 wt % NaCl solution. Interestingly, the created scratch self-healed, and the micro-crack diminished after 7 days of immersion in saline water. The aggressive saline solution attack through the micro-crack, leading to the PEI corrosion inhibitor leaching from the MSPs. Then, the dissolved PEI adsorbed on the uncoated steel and inhibited the corrosion process. As pH values vary from the neutral, both the MSPs and the PEI possess the same charge (positive at pH < 6 and negative at pH > 6).^44^ Accordingly, faster release of PEI is expected due to the higher electrostatic repulsion forces. Based on the results, the corrosion inhibitor (PEI) is released in response to the pH variation in an aggressive atmosphere.^44^

#### 3.6.2 EIS Analysis

**Figure 7:**
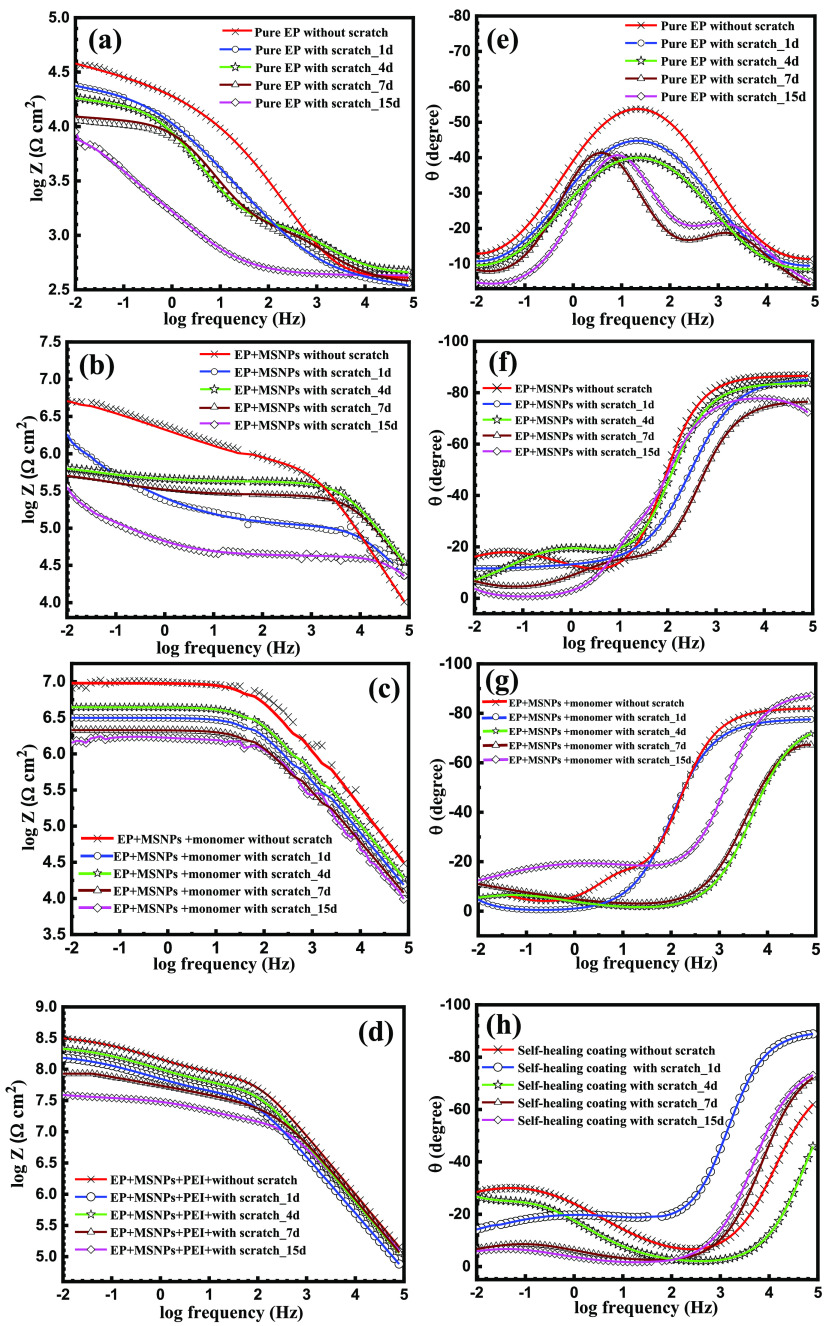
Bode plots of (a) pure EP coatings, (b) EP coatings after addition of MSPs, and (c,d) EP coatings after the addition of monomer-loaded MSPs-EP and monomer-loaded MSPs-PEI with and without a scratch after immersion in 3.5 wt % NaCl solution for various periods and their corresponding phase angle plots (e–h).

**Figure 8:**
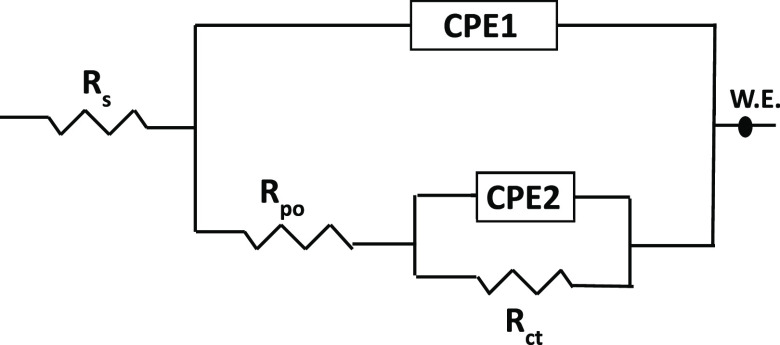
Equivalent circuit used to fit the Bode and phase angle plots of the EP coatings.

EIS was conducted to explore the anti-corrosion properties of the as-fabricated EP coatings. Figure 7 shows the EIS Bode plots of the pure EP coatings, EP + MSPs, and EP + MSPs with MSPs loaded with the PEI corrosion inhibitor after immersion for different periods of 1, 4, 7, and 15 days in saline water (3.5 wt % NaCl solution). Table 1 shows the derived electrochemical parameters utilizing the equivalent circuit in Figure 8, where *R*_sol_, *R*_ct_, and *R*_po_ represent the solution resistance, charge transfer resistance, and pore resistance, respectively. CEP1 is linked to coating capacitance, and its variation is expected to reflect the formation of conductive paths due to electrolyte uptake. At low frequency, CPE2 is ascribed to the capacitance of the double layer.

**Table 1:** EIS Electrochemical Factors of the EP Coatings at Various Compositions after Immersion in Saline water for 15 Days

sample	time (day)	*R*_po_ (kΩ cm^2^)	*R*_ct_ (kΩ cm^2^)	CPE1 (nF cm^–2^ s^α–1^)	*n*1	*C*_dl1_ (nF cm^–2^)	CPE2 (nF cm^–2^ s^α–1^)	*n*2	*C*_dl1_ (nF cm^–2^)
EP without scratch	1	8.2	31.6	56.2	0.786	35	30	0.708	27
EP with scratch	1	3	19.9	89	0.643	34	78	0.644	34
	4	0.7	15.8	248	0.567	121	201	0.631	133
	7	0.2	11.2	362	0.652	223	285	0.642	218
	15	0.1	7.9	761	0.631	565	480	0.617	554
EP + MSPs without scratch	1	1.7 × 10^3^	5.7 × 10^3^	14	0.721	5.2	12	0.752	5
EP + MSPs with scratch	1	0.3 × 10^3^	1.7 × 10^3^	60	0.634	16	45	0.677	13
	4	0.08 × 10^3^	0.6 × 10^3^	78	0.671	17	58	0.702	14
	7	0.06 × 10^3^	0.5 × 10^3^	102	0.635	18	88	0.653	16
	15	0.01 × 10^3^	0.3 × 10^3^	130	0.628	19	89	0.702	18
EP + monomer loaded MSPs without scratch	1	2.1 × 10^3^	7.1 × 10^3^	11	0.712	3.9	9	0.709	3.2
EP + monomer loaded MSPs with scratch	1	0.9 × 10^3^	3.2 × 10^3^	37	0.688	14	31	0.643	8.6
	4	1.2 × 10^3^	4.3 × 10^3^	31	0.675	11	25	0.633	6.8
	7	0.2 × 10^3^	2.1 × 10^3^	44	0.665	13	36	0.629	8.1
	15	0.1 × 10^3^	1.4 × 10^3^	57	0.655	15	49	0.618	9.3
EP + PEI loaded MSPs without scratch	1	59 × 10^3^	340 × 10^3^	2.1	0.706	1.7	2	0.7	0.7
EP + PEI loaded MSPs with scratch	1	62 × 10^3^	151 × 10^3^	4.2	0.641	3.2	10	0.631	1.8
	4	72 × 10^3^	199 × 10^3^	3.1	0.621	2.1	6	0.732	1.4
	7	40 × 10^3^	72 × 10^3^	6.1	0.625	3.6	18	0.682	3.1
	15	1 × 10^3^	40 × 10^3^	8.3	0.628	4.1	24	0.672	3.7

In the equivalent circuit, the capacitive element was substituted with a constant-phase element (CPE), which is used to simulate deviations from a non-ideal capacitive behavior. The double-layer capacitance (*C*_dl_) was calculated using the following formula.^34,45^
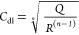
1where *R* is assigned to the charge transfer resistance (*R*_ct_) or the pore resistance (*R*_po_) and *Q* and *n* are the CPE constant and CPE exponent, respectively. When *n* = 1, then the CPE becomes equivalent to the ideal capacitor, and when *n* = 0, the CPE becomes equivalent to the resistor.

From Table 1, it can be noticed that the corrosion resistance of the scratched pure EP markedly decreased from 31.6 to 7.9 kΩ cm^2^ after 15 days of immersion due to the increase in water uptake by the coating surface. This low impedance modulus of EP at low frequency |*Z*_0.01 Hz_| was credited to the occurrence of defects that easily facilitate the ingress of hydrated Cl^–^ species from the front of fault through the osmotic pressure. Generally, corrosion in the EP coating can lead to (i) the increase in the pH values to 9 underneath the film because of oxygen reduction and formation of OH^–^ ions and/or (ii) the decline in the pH values to ∼4, owing to hydrolysis of ferrous ions. In both cases, the adhesion of the coating/metal interface will be damaged and subsequently increase the corrosion rate, while the addition of MSPs to the scratched EP coating increased the charge transfer resistance (*R*_ct_) to 1.7 MΩ cm^2^, which could be ascribed to the blockage of the defects existing in the EP coating by the inactive MSPs. Consequently, the permittivity of the aggressive ions (Cl^–^) to attack the metal surface was alleviated. Under this condition, *C*_dl1_ and *C*_dl2_ lessened from 565 and 554 nF cm^–1^ of the pure EP coating to 19 and 18 nF cm^–1^ for EP + MSPs after 15 days of immersion. It is noteworthy that the corrosion resistance moderately improved by loading the MSPs with the EP monomer compared to the unloaded ones. The *R*_ct_ increased to 4.3 MΩ cm^2^ after 4 days of immersion, revealing that the monomer-loaded MSP coating successfully self-healed the scratch; however, the corrosion resistance gradually diminished to 1.4 MΩ cm^2^ after 15 days of immersion. Interestingly, the self-healing coating loaded with the inhibitor before and after scratching displayed the highest corrosion resistance of 340 and 151 MΩ cm^2^, respectively. Noteworthy, the *R*_ct_ of the scratched smart coating increased to 199 MΩ cm^2^ after 4 days of immersion in comparison to the first day of immersion. Subsequently, the *C*_dl1_ and *C*_dl2_ decreased from 52 and 12 nF cm^–1^ to 46 and 6.4 nF cm^–1^, respectively. In addition, the *R*_ct_ of the smart coating gradually reduced after 7 and 15 days of immersion to 72 and 40 MΩ cm^2^, respectively. The higher corrosion resistance of the smart coating could be accredited to (i) the release of the mixed-type corrosion inhibitor (PEI), which can reduce both cathodic and anodic reactions on the steel surface,^46^ (ii) formation of an adsorbed layer of the PEI corrosion inhibitor on the steel surface as a result of the reaction of PEI with the generated metal ions (Mn^+^) in the anode area, leading to the construction of indissoluble hydroxides (OH^–^), which subsequently deposited as an insoluble layer on the steel surface, (iii) the self-healing effect as a result of the interaction between the released PEI inhibitor and EP monomer from the MSPs, leading to recovery of the scratched area of the modified coating, and/or (iv) the inorganic MSPs that act as inert charge carriers, which are generally added to primers and coatings to enhance their barrier properties against corrosion.^47^ The corrosion resistance of the as-prepared self-healing coating was compared with that of different coating systems, as shown in Table 2.

**Table 2:** Comparison of the Corrosion Resistance of the as-Prepared Self-Healing with Variable Coatings Systems

sr. no	coating structure	corrosion inhibitor	total time of immersion	corrosion resistance (Ω cm^2^)	refs
1	EP + PEI-loaded MSPs with scratches	PEI	15 d	4 × 10^7^	this work
2	5 wt % MSN-BTA@PDEAEMA	BTA	97 h	6.6 × 10^4^	(48)
3	EP graphene oxide (GO)/SiO_2_-based nanocontainer-loaded BTA	BTA	48 d	∼3.4 × 10^9^	(49)
4	5 wt % SiO_2_@BTA-modified PDMS coating	BTA	360 h	∼4.5 × 10^7^	(50)
5	EP resin primer doped with BTA@MSNs-COOH-PEI (12 wt % nanocontainers)	BTA	28 d	3.4 × 10^5^	(51)
6	graphene oxide (GO) and BTA-loaded mesoporous silica nanoparticles (BTA/MSNs)	BTA	30 d	1.4 × 10^7^	(52)
7	EP/mesoporous silica/sodium molybdate	sodium molybdate	56 d	1.1 × 10^6^	(16)

**Figure 9:**
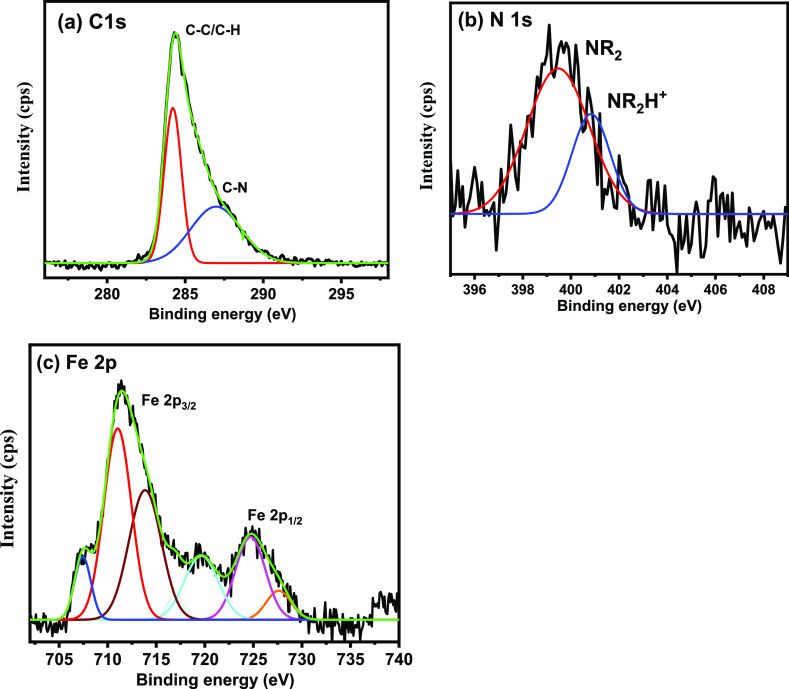
. High-resolution XPS spectra of (a) C 1s, (b) N 1s, and (c) Fe 2p after removing the self-healing EP coating immersed in 3.5 wt % NaCl for 15 days.

Figure 9 shows the high-resolution XPS spectra after removing the self-healing coating to explore the release of the corrosion inhibitor on carbon steel after immersion for 15 days in 3.5 wt % NaCl. The results revealed the adsorption of the PEI on carbon steel due to the drop in the pH value at the coating/metal interface, leading to protonation of the amine group, triggering the release of the corrosion inhibitor. It is noteworthy that the peaks positioned at 284.2 and 286.9 eV are linked to C–C/C–H bonds and C–N bonds of PEI, respectively. Deconvolution of N 1s high-resolution spectra gives mainly uncharged amine nitrogen peaks at low BE (−NR_2_ at 399.3 eV) and protonated amine groups located at almost +1.5 eV higher BE 400.8 eV; see Figure 9b. Fe 2p spectra in Figure 9c are deconvoluted into six peaks. In fact, the interpretation of Fe 2p spectra is complex due to the presence of iron (Fe) in variable oxidation states of Fe^0^, Fe^2+^, Fe^3+^, and satellites of Fe^3+^ ions.^53^ The (Fe 2p_3/2_ XPS spectrum at high resolution involves four bands at 707.3 eV of the metallic iron and 710.7 eV for Fe^3+^ of Fe_2_O_3_/FeOOH; however, the peak positioned at 713.8 eV is credited to a mixture of (Fe^2+^ and Fe^3+^), in different forms of iron(II) oxide (FeO), iron(II) hydroxide Fe(OH)_2_, iron(III) hydroxide Fe(OH)_3_, FeOOH, iron(III) oxide (Fe_2_O_3_), and magnetite (Fe_3_O_4_).^54^ The shake-up phenomenon observed at 714.3 and 719.8 eV is accredited to Fe^2+^ and Fe^3+^, respectively. The spectrum of the Fe 2p_1/2_ peaks at BE of 724.6 and 727.7 eV can be ascribed to Fe_2_O_3_ and FeOOH, respectively.^55^

#### 3.6.3 Localized Electrochemistry Testing

**Figure 10:**
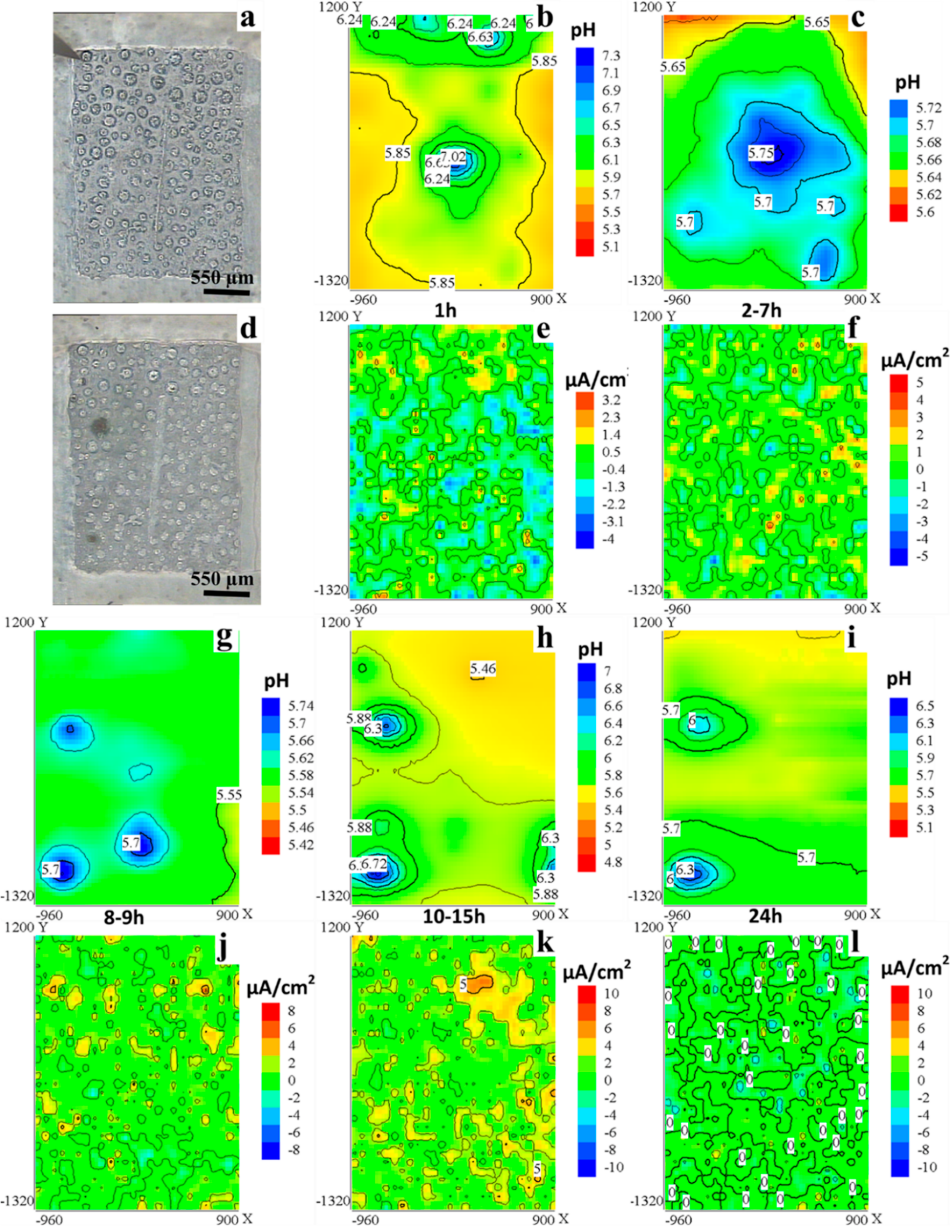
(a,d) Optical micrographs of EP coating loaded with MSPs at the first moment of immersion and after 24 h of immersion and pH distributions corresponding to (b) 1, (c) 2–7, (g) 8–9, (h) 10–15 , and (i) 24 h. Current density distributions corresponding to (e) 1, (f) 2–7, (j) 8–9, (k) 10–15, and (l) 24 h. *X* and *Y* correspond to the coordinates of the scan in μm.

EP coatings containing MSPs show the corrosion activity since the start of immersion, as evidenced in Figure 10. The SVET was able to identify a hint of cathodic activity after 8–10 h of immersion, correlated to slightly alkaline pH, see Figure 10g–j. Although, from 10 to 15 h of immersion, very weak anodic activity correlated to a small pH decrease was detected by SIET in the corresponding area Figure 9h–k. The activity in the other time intervals was detected solely by SIET, while the variation of current density was in a frame of the noise level. This can be explained as a combination of three possible factors: (i) the SIET microelectrode is positioned closer to the surface than the SVET microprobe and has a thinner tip, which makes it more sensitive to any slight change in the local pH, (ii) corrosion process occurs; however, the ions produced due to the corrosion process are trapped by inhibiting species present in the coating (e.g., amines, which are known to form complexes with iron ions), and (iii) propagation of the corrosion process underneath the coating, including both—anodic and cathodic processes.

Furthermore, it was noticed that alkalinization was greatly related to circular features and MSP agglomerates in the coating and not related to the artificial scratch (see p. 10). It suggests that MSP coatings show the corrosion activity at start of immersion and anodic activity is observed after 10–15 h of immersion, which decreases the pH to ∼pH 5. Such an event would be possible during interaction with H_2_O by means of hydrogen bonds and reversible equilibrium processes, exemplified using reactions
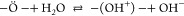
2
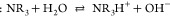
3

This process may contribute to creating a layer of OH^–^ “connected” to the agglomerates of MSPs via “O” atoms. Such hydroxide anions would be at the same time in the solution and weakly bonded to the MSPs. Hypothetically, the existence of the same type of layers would be enabled in the case of other compounds containing elements with high electronic negativity (e.g., “N” in amines). A simpler explanation would imply permeability of the coating and propagation of the corrosion process underneath the coating while releasing a small amount of OH^–^ through the active sites. The evidence of electrolyte permeability and accumulation was observed as MSPs do not provide better pore resistance due to the unavailability of the corrosion inhibitor. It was observed that under the EIS study, the capacitance of coating increases (CPE1) due to electrolyte penetration through the coating.

**Figure 11:**
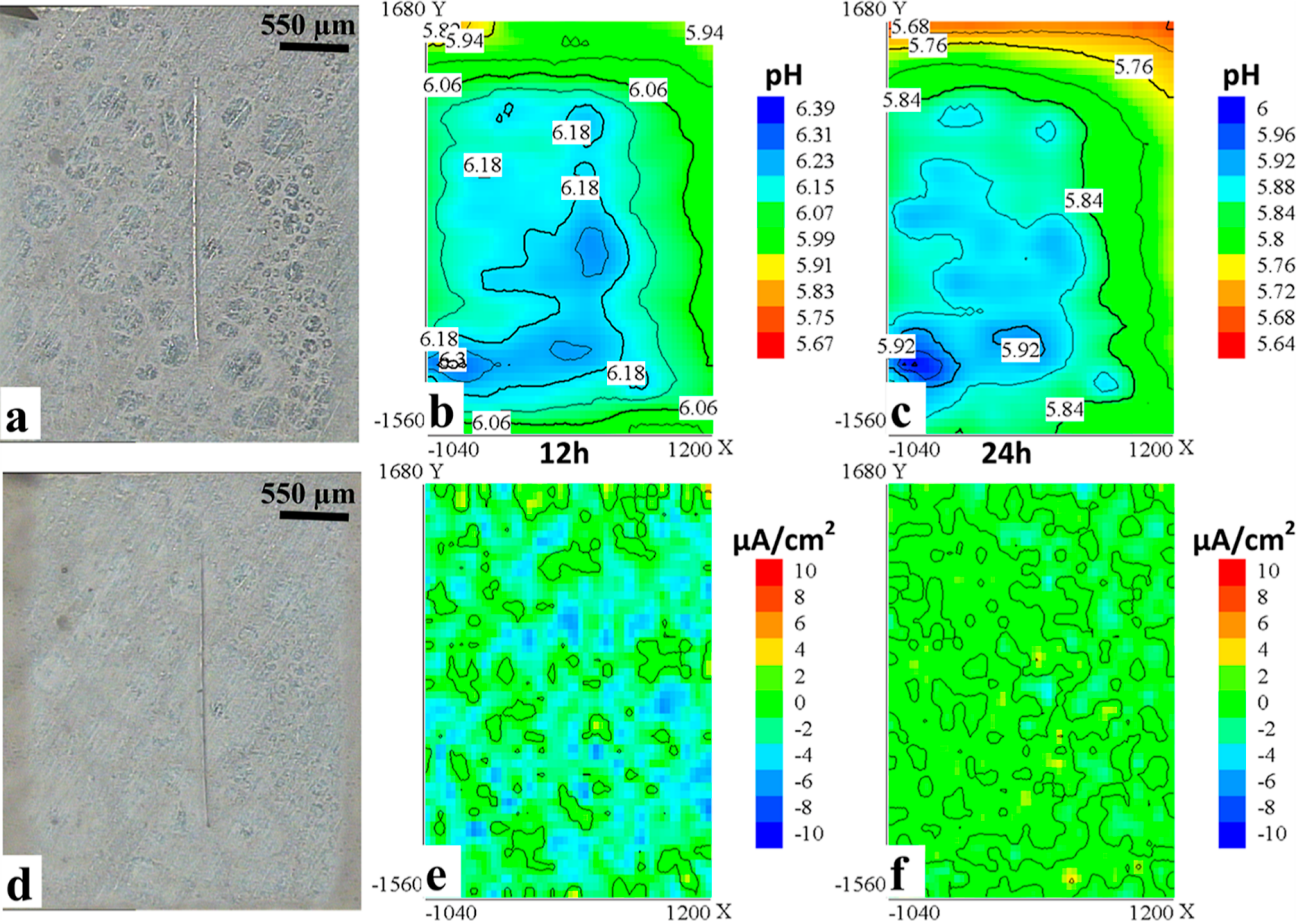
(a–d) Optical micrographs of the coating modified with the mixture (MSPs-Ep monomers + MSPs-PEI) at the first moment of immersion and after 24 h of immersion and pH distributions corresponding to (b) 12 h and (c) 24 h. Current density distributions correspond to (e) 12 h and (f) 24 h. *X* and *Y* correspond to the coordinates of the scan in μm.

**Figure 12:**
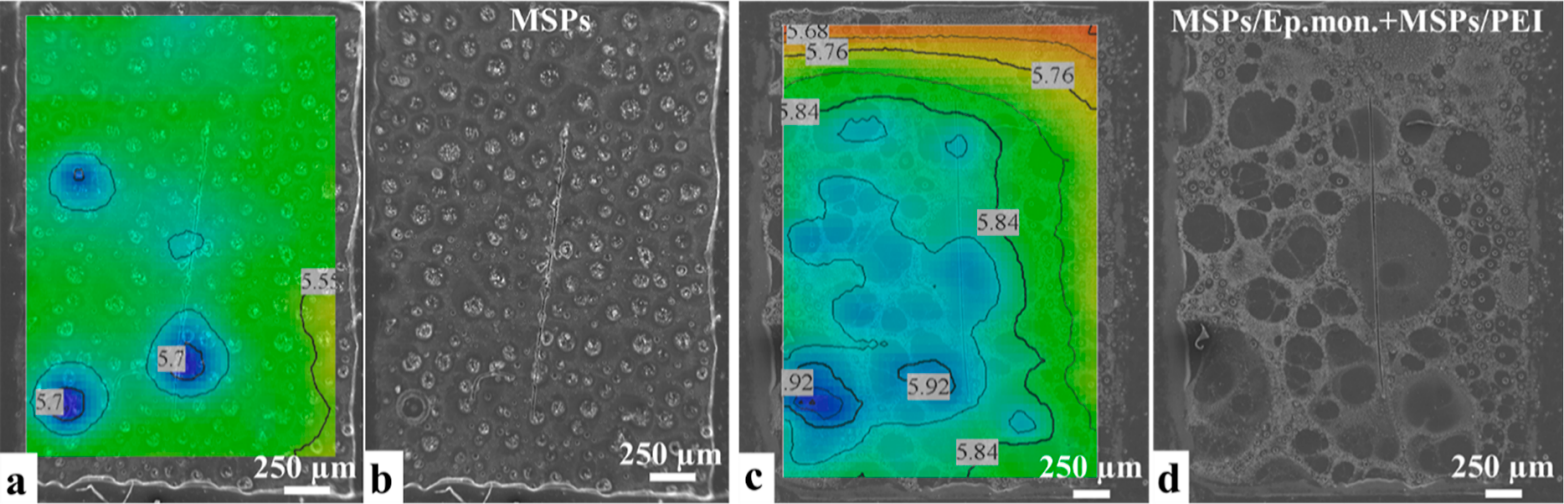
SEM micrograph (a) and its overlap with the typical pH distribution (b) obtained for the coated sample with unloaded MSPs and SEM micrograph (c) and its overlap with the typical pH distribution (d) obtained for the coated sample with the mixture (MSPs-Ep monomers + MSPs-PEI).

The general behavior of the protective coating loaded with the mixture (MSPs-Ep monomers + MSPs-PEI) is illustrated by the representative pH and current density distributions in Figure 11. The sensitivity of SVET was not sufficient to detect any reproducible corrosion activity during the entire immersion time. Similar to the coating with non-inhibited MSPs, SIET was able to record a certain alkalinization above the surface, strongly correlated to the circular features also present in the coating (Figure 12). pH values above the “active” sites remained 5.7–6.2 during the entire immersion time. No damage was visible in optical micrographs after 24 h of immersion. Alkalinization without corresponding changes in current density and evident damage could be assigned to release of the loaded components on MSPs.

**Figure 13:**
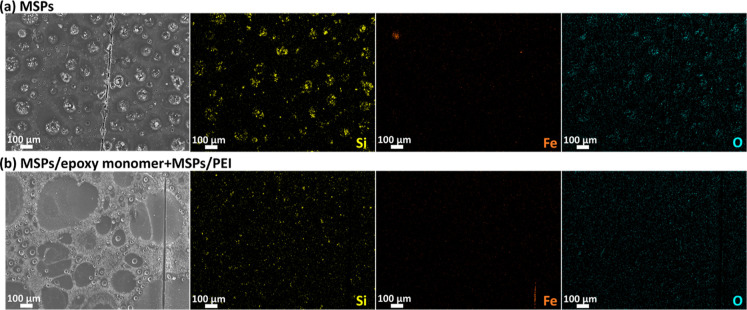
SEM/EDS assessment of the scratched samples after 24 h of immersion in 0.05 M NaCl. (a) SEM micrograph and corresponding elemental distributions of Si, Fe, and O obtained in the case of the coating modified with unloaded MSPs and (b) SEM micrograph and corresponding elemental distributions of Si, Fe, and O obtained in case of the coating modified with MSPs-Ep monomers + MSPs-PEI.

Figure 13b demonstrates that a part of the scratch remained active, exposing the steel substrate. However, the corrosion process was inhibited (possibly due to the presence of PEI), and no relevant corrosion propagation was identified near the exposed area. Remarkably, the agglomeration of MSPs in the coating was significantly reduced when a mixture of MSPs (loaded with EP monomers + PEI) was used, Figure 13. This means that this coating is slightly more protective and can provide good corrosion inhibition.

#### 3.6.4 Self-Healing Mechanism

**Figure 14:**
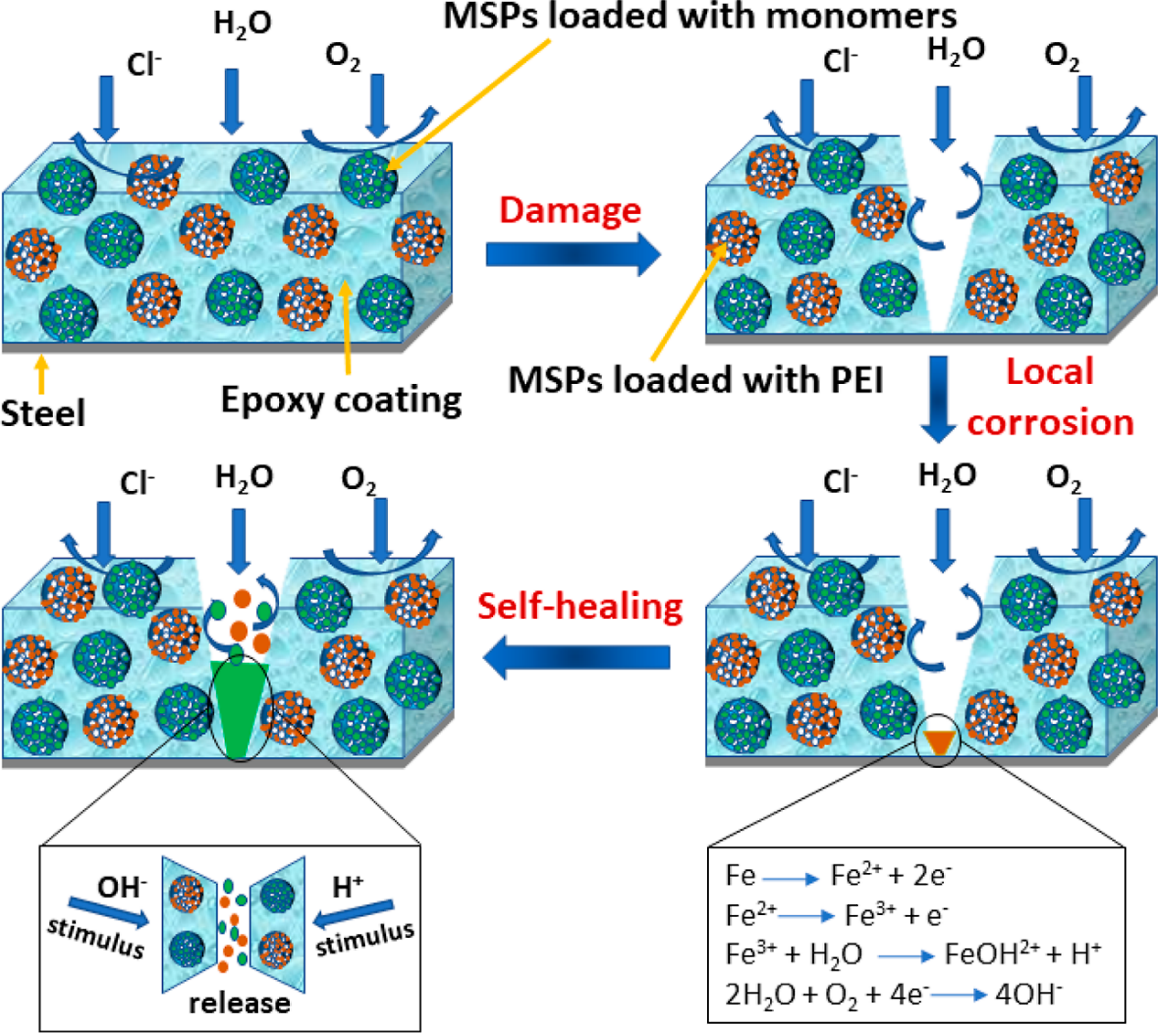
Proposed self-healing regime of the scratched EP coatings PEI-loaded mesoporous silica.

Coating imperfections, such as micropores and microcracks, can take place during coating preparation, which enable the hydrated aggressive ions like Cl^–^ to diffuse into the epoxy coating matrix, consequently starting the steel deterioration. This could lead to the construction of microgalvanic couples in the localized zone, resulting in a drop of the local pH value in the anodic area because of the hydrolysis of metal ions. Nevertheless, the generated OH^–^ species from the oxygen reduction reaction adjacent to the electrodes increase the local alkalinity. The loaded PEI molecules are released from the MSPs and form a protection film adsorbed on the unprotected steel, which eventually decreases the corrosion attack. The PEI obeys the Langmuir adsorption isotherm, adopting a physichemisorption mechanism.^46^ The electrostatic interactions between charged metal surfaces and the charged PEI molecules are a reason for physisorption. However, the chemisorption is due to the transfer of the π electrons from N atoms of PEI to the vacant 3d orbitals of iron, which leads to the π-d complex. The difference in the local pH values could accelerate this process. The schematic diagram of the self-healing regime is presented in Figure 14.

## 4 Conclusions

MSPs used a carrier for loading with PEI and Ep monomers, which further incorporated into coating an additive for corrosion protection of steels. FTIR and TGA results confirm the loading of PEI and Ep monomers with MSPs. Electrochemical properties of the developed self-healing coatings show corrosion inhibition of the coating. The EIS analysis concludes that smart coatings of PEI-loaded MSPs with the addition of MSPs-Ep monomers demonstrate improved anti-corrosive properties in comparison to a coating containing MSPs only. Low activity was detectable in the pH maps during the entire immersion time. SVET did not detect significant ionic flows in the sample with the modified coating (MSPs-Ep monomers + MSPs-PEI), and only short intervals of shallow cathodic and anodic activity were observed, which were attributed to the sufficient release of the PEI corrosion inhibitor, which mitigates the corrosion process and recovers the micro-cracks of the scratched coatings. Moreover, addition of Ep monomers helps in self-healing during 1 week of immersion.).
